# Impact of post-hepatectomy liver failure on morbidity and short- and long-term survival after major hepatectomy

**DOI:** 10.1093/bjsopen/zrac097

**Published:** 2022-07-16

**Authors:** Ruth Baumgartner, Stefan Gilg, Bergthor Björnsson, Kristina Hasselgren, Poya Ghorbani, Christina Sauter, Per Stål, Per Sandstöm, Ernesto Sparrelid, Jennie Engstrand

**Affiliations:** Division of Surgery, Department of Clinical Science, Intervention and Technology, Karolinska Institutet, Karolinska University Hospital, Stockholm, Sweden; Division of Surgery, Department of Clinical Science, Intervention and Technology, Karolinska Institutet, Karolinska University Hospital, Stockholm, Sweden; Department of Biomedical and Clinical Sciences, Division of Surgery, Linköping University, Linköping, Sweden; Department of Biomedical and Clinical Sciences, Division of Surgery, Linköping University, Linköping, Sweden; Division of Surgery, Department of Clinical Science, Intervention and Technology, Karolinska Institutet, Karolinska University Hospital, Stockholm, Sweden; Division of Surgery, Department of Clinical Science, Intervention and Technology, Karolinska Institutet, Karolinska University Hospital, Stockholm, Sweden; Division of Hepatology, Department of Medicine, Huddinge, Karolinska Institutet, Stockholm, Sweden; Department of Biomedical and Clinical Sciences, Division of Surgery, Linköping University, Linköping, Sweden; Division of Surgery, Department of Clinical Science, Intervention and Technology, Karolinska Institutet, Karolinska University Hospital, Stockholm, Sweden; Division of Surgery, Department of Clinical Science, Intervention and Technology, Karolinska Institutet, Karolinska University Hospital, Stockholm, Sweden

## Abstract

**Background:**

Post-hepatectomy liver failure (PHLF) is one of the most serious postoperative complications after hepatectomy. The aim of this study was to assess the impact of the International Study Group of Liver Surgery (ISGLS) definition of PHLF on morbidity and short- and long-term survival after major hepatectomy.

**Methods:**

This was a retrospective review of all patients who underwent major hepatectomy (three or more liver segments) for various liver tumours between 2010 and 2018 at two Swedish tertiary centres for hepatopancreatobiliary surgery. Descriptive statistics, regression models, and survival analyses were used.

**Results:**

A total of 799 patients underwent major hepatectomy, of which 218 patients (27 per cent) developed ISGLS-defined PHLF, including 115 patients (14 per cent) with ISGLS grade A, 76 patients (10 per cent) with grade B, and 27 patients (3 per cent) with grade C. The presence of cirrhosis, perihilar cholangiocarcinoma, and gallbladder cancer, right-sided hemihepatectomy and trisectionectomy all significantly increased the risk of clinically relevant PHLF (grades B and C). Clinically relevant PHLF increased the risk of 90-day mortality and was associated with impaired long-term survival. ISGLS grade A had more major postoperative complications compared with no PHLF but failed to be an independent predictor of both 90-day mortality and long-term survival. The impact of PHLF grade B/C on long-term survival was no longer present in patients surviving the first 90 days after surgery.

**Conclusions:**

The presently used ISGLS definition for PHLF should be reconsidered regarding mortality as only PHLF grade B/C was associated with a negative impact on short-term survival; however, even ISGLS grade A had clinical implications.

## Teaser

The aim was to assess the ISGLS criteria for post-hepatectomy liver failure (PHLF) in a cohort of patients with major hepatectomy. The presently used ISGLS definition for PHLF should be reconsidered regarding mortality as only PHLF grade B/C was associated with a negative impact on short-term survival.

## Introduction

Despite technical improvements and careful patient selection, the development of post-hepatectomy liver failure (PHLF) and the associated high risk of postoperative mortality remains a major concern after hepatectomy^1,2^.

Several definitions of PHLF are used today, thus the lack of uniformity affects the interpretation of incidence and related mortality. In 2005, Balzan *et al*. defined PHLF according to the ‘50–50 criteria’ and found it to be associated with a 60-day mortality as high as 59 per cent^3^. Later, Mullen *et al*. found peak bilirubin higher than 7 mg/dl to be a better predictor of 90-day mortality^4^. Subsequently, the International Study Group of Liver Surgery (ISGLS) defined PHLF as an increased international normalized ratio (INR) and simultaneous hyperbilirubinaemia on or after postoperative day five^5^. The severity of PHLF according to ISGLS is graded, where grade B and C require a change of the patient’s clinical management and are associated with mortality rates of 13 per cent and 54 per cent respectively^5,6^.

As ISGLS grade A does not imply a change of clinical management, its clinical impact on both short- and long-term survival can be questioned. A recent study on a cohort of major hepatectomies found that ISGLS grade A PHLF was not associated with 90-day mortality^7^. Whether ISGLS grade A has impact on long-term survival remains to be shown.

While it is well established that PHLF presents a high risk for mortality in the immediate postoperative interval, less is known about its effect on long-term survival, and the results are partly contradictory. Ito *et al*. found PHLF to be an independent predictor of decreased long-term survival after hepatectomy for colorectal cancer liver metastases (CRLMs)^8^. Joechle *et al*. on the other hand, did not find an association between ISGLS-defined PHLF or ISGLS grade B or C PHLF and long-term survival, whereas peak bilirubin was associated with both short-term morbidity and worse long-term survival^9^.

Hence, this study aimed to assess the impact of the ISGLS definition of PHLF as well as the ‘50–50 criteria’ on both short- and long-term survival after major hepatectomy. Secondary aims were to identify risk factors for 90-day mortality and the development of PHLF in this cohort.

## Methods

### Study population and data collection

The study population included all consecutive patients that underwent elective major hepatectomy at two Swedish tertiary centres for hepatopancreatobiliary surgery (Karolinska University Hospital, Stockholm and Linköping University Hospital, Linköping) between January 2010 and December 2018. Major hepatectomy was defined as resection of three or more liver segments according to Couinaud’s classification and trisectionectomy was defined as resection of five or more liver segments^10^. The study was conducted in accordance with the Declaration of Helsinki and approved by the local ethics committees. Patients younger than 18 years, with liver resection in the setting of associating liver partition, and portal vein ligation for staged hepatectomy, trauma, liver transplant donation, and patients with previous liver transplantation were not included in the study cohort.

All patients were discussed at the weekly hepatobiliary multidisciplinary team meeting consisting of liver surgeons, radiologists, and medical oncologists. Recommended treatment strategy was based on current available guidelines for each primary tumour^11–13^ taking patient fitness for surgery and co-morbidity into account and assessing technical resectability based on the principles of performing complete resection with preserved sufficient future liver remnant (FLR) with adequate inflow and outflow. Oncological systemic treatment was administered before and after surgery according to national standards.

All patients underwent preoperative clinical and laboratory examination according to the institution’s standard. CT was performed routinely and was completed with MRI or ultrasound if necessary. To determine FLR a CT volumetric study was performed and generally an FLR of 25–30 per cent or more of the total estimated liver volume^14^ was considered necessary to proceed with major hepatectomy depending mainly on assessment of the functional quality of the FLR. If an insufficient FLR was present before surgery, portal vein occlusion (embolization or ligation) was performed with reassessment of the FLR after 2–4 weeks. During hepatectomy, intraoperative ultrasound was utilized to verify tumour location and contribute to a parenchyma-sparing approach. Parenchymal dissection was mainly performed with a cavitron ultrasonic surgical aspirator and occasionally, combined blunt-clamp dissection and LigaSure ligation or water-jet dissection. An intermittent Pringle manoeuvre, up to 15 min of occlusion followed by 5 min of releasing, was applied at the time of liver transection in selected cases.

After surgery, all patients were assessed daily and discharged when mobile, tolerating food intake, and under adequate pain control without signs of untreated complications. A postoperative visit to the outpatient clinic was routinely performed after 4 weeks with follow-up CT every 3 to 6 months, depending on the primary tumour, to detect recurrent disease.

### Definition of study outcomes

Data on patient, tumour, and procedure-related characteristics were retrospectively collected from hospital medical records. The underlying tumour was defined by histopathological diagnosis. Postoperative serum bilirubin, prothrombin time, and INR were recorded to categorize the occurrence of PHLF according to the ‘50–50 criteria’ proposed by Balzan *et al*. and the grading system established by the ISGLS. ISGLS grade B and C were summarized as ‘clinically relevant’ as they require a change of clinical management^3,5,15^. Postoperative haemorrhage and bile leakage were defined and graded according to the consensus definitions by the ISGLS^16,17^. The number of transfused packed red blood cells was summarized for the day of surgery and postoperative day 1.

Postoperative 90 day-morbidity was described according to the Clavien–Dindo classification^18^, with the single highest complication per patient graded. Grade I–II were summarized as minor morbidity and grade IIIa and higher as major complications. Early postoperative mortality was analysed 90 days after surgery. Overall survival (OS) was calculated from date of surgery to death or censored in February 2021 if the patient was still alive.

### Statistical analysis

Categorical data were presented as absolute frequencies and percentages and compared using the chi-squared test or Fisher's exact test; the latter used for sample sizes smaller than 10. Continuous data were presented as median (non-normally distributed data) with minimum and maximum, and differences were tested with a Wilcoxon rank sum test or Kruskal–Wallis equality-of-populations rank test.

Independent risk factors for PHLF and 90-day mortality were determined by logistic regression and presented as odds ratios (OR) with 95 per cent confidence interval (c.i.). Factors with a *P* value <0.100 in univariable analysis were included in multivariable model.

The median follow-up was assessed with a reverse Kaplan–Meier method^19^. Survival curves were generated using the Kaplan–Meier method and compared using the log rank test. Independent prognostic factors for survival were determined by Cox proportional hazards regression model and factors with *P* < 0.100 in the univariable analysis were included in the multivariable model and reported as HR with associated 95 per cent confidence interval. The impact of PHLF on long-term survival was analysed in the entire cohort and separately after exclusion of the patients that died within 90 days of major hepatectomy. Statistical significance was set at a two-sided α level of 0.05. All statistical analyses were performed using STATA 15.0 (StataCorp, College Station, Texas, USA).

## Results

A total of 829 patients underwent major hepatectomy during the study interval. After exclusion of the two patients with lethal intraoperative complications and a further 28 patients due to unknown primary tumour on the pathology report, the remaining 799 patients constituted the study cohort. Differences in pre- and postoperative characteristics depending on PHLF grade are outlined in *Tables 1* and *2*.

**Table 1:** Preoperative characteristics in patients with or without post-hepatectomy liver failure with post-hepatectomy liver failure defined according to the International Study Group of Liver Surgery and according to ‘50–50 criteria’ by Balzan *et al.*

	All patients	PHLF according to ISGLS				PHLF according to ‘50-50 criteria’		
	*n* = 799	ISGLS 0 *n* = 581	ISGLS A *n* = 115	ISGLS B/C *n* = 103	*P* *,†	No PHLF *n* = 763	PHLF *n* = 36	*P* †
**Patient characteristics**								
Age (years), median (range)	65 (18–85)	66 (20–85)	63 (18–83)	66 (26–82)	0.149‡	66 (18-85)	63.5 (26–80)	0.386‡
Sex ratio (M:F)	438:55	293:50	82:71	63:61	<0.001	411:54	27:75	0.013
Diabetes	106 (13)	73 (13)	11 (10)	22 (21)	0.026	99 (13)	7 (19)	0.310
Pulmonary disease	78 (10)	61 (11)	12 (11)	5 (5)	0.185	76 (10)	2 (6)	0.567
Cardiovascular disease	330 (41)	247 (43)	35 (31)	48 (47)	0.039	314 (41)	16 (44)	0.705
**Tumour/liver characteristics**								
Cirrhosis	27 (3)	11 (2)	3 (3)	13 (13)	<0.001	22 (3)	5 (14)	0.005
Tumour type								
CRLM	418 (52)	312 (54)	64 (56)	42 (41)	<0.001	402 (53)	16 (44)	<0.001
HCC	62 (8)	46 (8)	11 (10)	5 (5)		59 (8)	3 (8)	
pCCC	94 (12)	53 (9)	15 (13)	26 (25)		86 (11)	8 (22)	
iCCA	68 (9)	57 (10)	7 (6)	4 (4)		67 (9)	1 (3)	
Gallbladder cancer	20 (3)	6 (1)	3 (3)	11 (11)		15 (2)	5 (14)	
Other malignant tumours	60 (8)	48 (8)	8 (7)	4 (4)		59 (8)	1 (3)	
Benign tumours	77 (10)	59 (10)	7 (6)	11 (11)		75 (10)	2 (6)	
**Preoperative tests**								
Bilirubin (μmol/l), median (range)	7 (0–290)	6 (0–58)	9 (3–123)	9 (2–290)	<0.001‡	7 (0–290)	9 (3–62)	0.008‡
INR, median (range)	1.0 (0.8–2.5)	1.0 (0.8–2.5)	1.0 (0.8–2.5)	1.0 (0.8–1.6)	0.074‡	1 (0.8–2.5)	1. (0.9–1.5)	0.024‡
Albumin (g/l), median (range)	35 (16–472)	35 (16–114)	35 (26–472)	34.5 (22–45)	0.191‡	35 (16–472)	36.5 (22–45)	0.312‡
AST (µkat/l), median (range)	0.56 (0.22–8.98)	0.53 (0.22–8.98)	0.59 (0.28–3.61)	0.67 (0.3–2.23)	<0.001‡	0.55 (0.22–8.98)	0.65 (0.3–2.1)	0.086‡
ALT (µkat/l), median (range)	0.49 (0.11–8.35)	0.45 (0.11–8.35)	0.53 (0.14–6.8)	0.76 (0.18–3.85)	<0.001‡	0.48 (0.11–8.35)	0.62 (0.19–3.85)	0.121‡

Values are *n* (%) unless otherwise indicated.

* *P* value refers to a comparison between all three groups.

† Categorical variables were compared with the chi-squared test or Fisher’s exact test as appropriate. Continuous variables were compared with the Wilcoxon rank sum test (two-group comparison) or Kruskal–Wallis equality-of-populations rank test (three-group comparison).

‡ Wilcoxon rank sum test or Kruskal–Wallis equality-of-populations rank test. PHLF, post-hepatectomy liver failure; ISGLS, International Study Group of Liver Surgery; CRLM, colorectal cancer liver metastases; HCC, hepatocellular carcinoma; pCCC, perihilar cholangiocarcinoma; iCCA, intrahepatic cholangiocarcinoma; INR, International Normalized Ratio; ALT, alanine aminotransferase; AST, aspartate aminotransferase.

**Table 2:** Intra- and postoperative parameters, including postoperative morbidity and mortality in patients with or without post-hepatectomy liver failure with post-hepatectomy liver failure defined according to the International Study Group of Liver Surgery or according to the ‘50-50 criteria’ by Balzan *et al.*

	All patients	PHLF according to ISGLS				PHLF according to ‘50–50 criteria’		
	*n* = 799	ISGLS 0 *n* = 581	ISGLS A *n* = 115	ISGLS B/C *n* = 103	*P* *,†	No PHLF *n* = 763	PHLF *n* = 36	*P* †
**Intraoperative factors**								
Portal vein occlusion	102 (13)	58 (10)	18 (16)	26 (25)	<0.001	92 (12)	10 (28)	0.006
Extent of liver resection								
Right hemihepatectomy	428 (54)	308 (53)	68 (59)	52 (50)	<0.001	409 (54)	19 (53)	0.005
Right trisectionectomy	172 (21)	105 (18)	23 (20)	44 (43)		157 (21)	15 (42)	
Left hemihepatectomy	135 (17)	116 (20)	15 (13)	4 (4)		133 (17)	2 (6)	
Left trisectionectomy	64 (8)	52 (9)	9 (8)	3 (3)		64 (8)	0	
Pringle manoeuvre	69 (9)	48 (8)	5 (4)	16 (16)	0.011	61 (8)	8 (22)	0.003
Prolonged operating time (>240 min)	491 (63)	339 (59)	66 (59)	86 (87)	<0.001	460 (61)	31 (89)	0.001
Operating time (min), median (range)	270 (85–792)	260 (95–735)	263 (85–592)	337 (145–792)	<0.001‡	268 (85–735)	383 (145–792)	<0.001‡
Multivisceral resection	50 (6)	35 (6)	5 (4)	10 (10)	0.239	47 (6)	3 (8)	0.487
Perioperative blood transfusion	251 (31)	168 (29)	26 (23)	57 (55)	<0.001	232 (30)	19 (53)	0.005
**Postoperative outcomes**								
Admitted to ICU within (24 h)	27 (3)	9 (2)	2 (2)	16 (16)	<0.001	17 (2)	10 (28)	<0.001
Post-hepatectomy haemorrhage§	62 (8)	29 (5)	8 (7)	25 (24)	<0.001	53 (7)	9 (25)	0.001
Post-hepatectomy bile leakage	173 (22)	115 (20)	26 (23)	32 (31)	0.037	161 (21)	12 (33)	0.082
**Morbidity/mortality**								
Postoperative complications								
None	25 (3)	17 (3)	8 (7)	0	<0.001	25 (3)	0	<0.001
Minor complications¶	456 (59)	374 (66)	58 (51)	24 (23)		448 (60)	8 (22)	
Major complications#	299 (38)	173 (31)	47 (42)	79 (77)		271 (37)	28 (78)	
Postoperative 90-day mortality	38 (5)	11 (2)	5 (4)	22 (21)	<0.001	27 (4)	11 (31)	<0.001

Values are *n* (%) unless otherwise indicated.

* *P* value refers to a comparison between all three groups.

† Categorical variables were compared using the chi-squared test or Fisher’s exact test as appropriate. Continuous variables were compared using the Wilcoxon rank sum test (two-group comparison) or Kruskal–Wallis equality-of-populations rank test (three-group comparison).

‡ Wilcoxon rank sum test or Kruskal–Wallis equality-of-populations rank test.

§ Definition and grading by the ISGLS.

¶ Minor complications only defined as Clavien–Dindo grade I–II within 90 days after surgery.

# Major complications defined as Clavien–Dindo grade III–V within 90 days after surgery. PHLF, post-hepatectomy liver failure; ISGLS, International Study Group of Liver Surgery; ICU, intensive care unit.

Some 218 patients (27 per cent) developed ISGLS-defined PHLF, including 115 patients (14 per cent) with ISGLS grade A, 76 patients (10 per cent) with ISGLS grade B and 27 patients (3 per cent) with grade C PHLF. When applying the definition by Balzan *et al*., 36 patients (5 per cent) fulfilled the ‘50–50 criteria’, of which 3 patients fulfilled the criteria for ISGLS PHLF grade A, 17 for grade B and 16 for grade C.

### Risk factors for PHLF

*Table S1* shows the association between patient, tumour, and intraoperative characteristics and clinically relevant PHLF (grade B and C). In the multivariable logistic regression analysis, cirrhosis (OR 45.31, 95 per cent c.i. 6.56 to 313.1), perihilar cholangiocarcinoma (OR 3.73, 95 per cent c.i. 1.37 to 10.18), gallbladder cancer (OR 6.39, 95 per cent c.i. 1.11 to 36.77), right-sided hemihepatectomy (OR 5.35, 95 per cent c.i. 1.37 to 20.84), trisectionectomy (OR 6.99, 95 per cent c.i. 1.74 to 28.06) and prolonged operating time (OR 3.72, 95 per cent c.i. 1.59 to 8.69) increased the risk of clinically significant PHLF, whereas pulmonary disease (OR 0.17, 95 per cent c.i. 0.03 to 0.83) was a protective factor.

### Postoperative morbidity, mortality, and risk factors for 90-day mortality

Major postoperative complications occurred in 299 patients (38 per cent) within 90 days of hepatectomy (*Table 2*). Patients with PHLF grade A suffered from major complications significantly more frequently than patients with no PHLF (42 per cent *versus* 31 per cent, *P* = 0.024, OR 1.61 with 95 per cent c.i. 1.06 to 2.44). Fulfilling the ‘50–50’ criteria was associated with major postoperative complications within 90 days of surgery in which 78 per cent had major complications *versus* 36 per cent in patients with no PHLF according to Balzan *et al*. (OR 6.11, 95 per cent c.i. 2.75 to 13.60, *P* < 0.001).

Overall 90-day mortality was 5 per cent (38 patients) and 21 per cent (22 patients) in clinically relevant PHLF (*Table 2*). Postoperative 90-day mortality was predicted by ISGLS clinically relevant PHLF (OR 7.26, 95 per cent c.i. 2.90 to 18.17), age above 65 years (OR 2.58, 95 per cent c.i. 1.08 to 6.14), gallbladder cancer (OR 8.82, 95 per cent c.i. 1.77 to 43.97), and perihilar cholangiocarcinoma (OR 3.71, 95 per cent c.i. 1.04 to 13.26), whereas ISGLS grade A PHLF and the extent of liver resection were not independent risk factors for 90-day mortality (*Table S2*). PHLF according to the ‘50–50 criteria’ was also significantly associated with 90-day mortality (OR 6.73, 95 per cent c.i. 2.41 to 18.79).

### Survival analysis and prognostic factors for long-term overall survival

The median follow-up interval after hepatectomy was 30 months (range 0.1–130 months, interquartile range (i.q.r.) 46 months) and median follow-up for patients who were alive at end of follow-up was 52 months (range 0.1–130 months, i.q.r. 56 months). Median estimated survival in the entire cohort undergoing major hepatectomy was 40 months (95 per cent c.i. 35 to 45 months). In patients with malignant diagnoses, the estimated 5-year OS rates according to the primary tumour were 36 per cent (95 per cent c.i. 31 to 41 per cent) for patients with CRLM, 51 per cent (95 per cent c.i. 37 to 64 per cent) for hepatocellular carcinoma (HCC), 30 per cent (95 per cent c.i. 21 to 40 per cent) for perihilar cholangiocarcinoma, and 22 per cent (95 per cent c.i. 12 to 33 per cent) for patients with intrahepatic cholangiocarcinoma. Patients with gallbladder cancer reached an estimated 2-year OS of 5 per cent (95 per cent c.i. 0.4 to 21 per cent) after major hepatectomy.

**Fig. 1:**
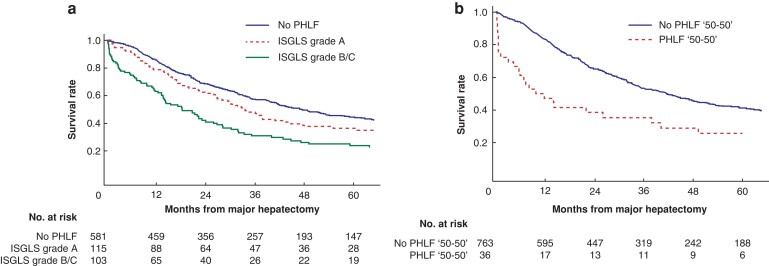
**a** Kaplan–Meier estimates of overall survival in patients with or without post-hepatectomy liver failure (PHLF) defined by the International Study Group of Liver Surgery (ISGLS). Grade A and grade B/C PHLF had a median survival and 5-year overall survival of 34 months (95 per cent c.i. 26 to 44 months) and 36 per cent for grade A and 18 months (95 per cent c.i. 14 to 26 month) and 24 per cent respectively, for grade B/C (log rank test, *P* = 0.006). No PHLF resulted in a median survival of 47 months (95 per cent c.i. 41 to 57 months) and 5-year overall survival of 45 per cent. **b** Kaplan–Meier estimates of overall survival in patients with or without PHLF according to the ‘50–50 criteria’ defined by Balzan *et al*. No PHLF had a median survival and 5-year overall survival of 42 months (95 per cent c.i. 35 to 47 months) and 41 per cent respectively, and PHLF according to the ‘50–50’ criteria had a median survival and 5-year overall survival of 10 months (95 per cent c.i. 4 to 38 months) and 26 per cent (log rank test, *P* < 0.001).

Median estimated survival in patients with ISGLS grade A and B/C was 34 months (95 per cent c.i. 26 to 44) and 18 months (95 per cent c.i. 14 to 26) respectively (*P* = 0.006), compared with 47 months (95 per cent c.i. 41 to 57) in patients without liver failure (*Fig. 1a*). Corresponding survival estimates when PHLF was defined by Balzan *et al*. are illustrated in *Fig. 1b*.

Multivariable Cox regression analysis identified PHLF grade B/C (HR 1.90, 95 per cent c.i. 1.32 to 2.71) as an independent risk factor of worse long-term survival, along with simultaneous multivisceral resection (HR 1.77, 95 per cent c.i. 1.01 to 3.09), gallbladder cancer (HR 7.28, 95 per cent c.i. 3.26 to 16.28), and portal vein occlusion (HR 1.63, 95 per cent c.i. 1.14 to 2.33), whereas ISGLS grade A PHLF (HR 1.25, 95 per cent c.i. 0.87 to 1.81) was not associated with impaired long-term survival. Malignant tumours (other than CRLM, cholangiocarcinoma, or HCC) (HR 0.34, 95 per cent c.i. 0.16 to 0.73) and benign tumours (HR 0.13, 95 per cent c.i. 0.05 to 0.35) were independently associated with a decreased risk of mortality (*Table S3*).

### Long-term survival in patients surviving 90 days after hepatectomy

**Fig. 2:**
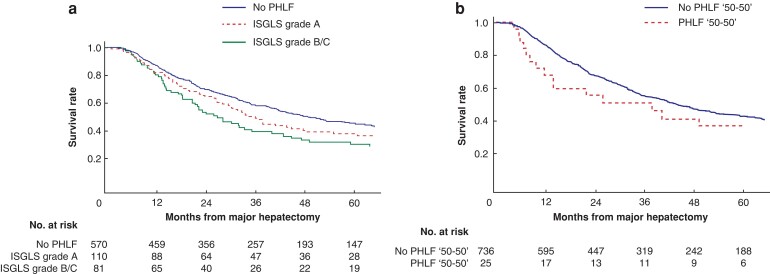
**a** Kaplan–Meier estimates of overall survival in patients surviving 90 days after hepatectomy according to post-hepatectomy liver failure (PHLF) defined by the International Study Group of Liver Surgery (ISGLS). Grade A and grade B/C PHLF had a median survival and 5-year overall survival of 36 months (95 per cent c.i. 28 to 48 months) and 38 per cent, respectively, for grade A and 27 months (95 per cent c.i. 21 to 40 months) and 30 per cent respectively, for grade B/C (log rank test, *P* = 0.213). No PHLF resulted in a median survival of 49 months (95 per cent c.i. 42 to 59 months) and 5-year overall survival of 45 per cent. **b** Kaplan–Meier estimates of overall survival in patients surviving 90 days after hepatectomy with or without PHLF according to the ‘50–50 criteria’ defined by Balzan *et al*. No PHLF had a median survival and 5-year overall survival of 44 months (95 per cent c.i. 39 to 51 months) and 43 per cent respectively, with corresponding survival estimates in PHLF of 38 months (95 per cent c.i. 12 to not reached) and 37 per cent respectively (log rank test, *P* = 0.237).

As shown in *Table S4*, when excluding the 38 patients who died within 90 days of hepatectomy, neither ISGLS grade A PHLF nor clinically relevant PHLF (grade B/C) was associated with impaired long-term survival in multivariable Cox proportional hazards regression model (HR 1.28, 95 per cent c.i. 0.97 to 1.70 and HR 1.30, 95 per cent c.i. 0.95 to 1.77 respectively). PHLF according to the ‘50–50 criteria’ (HR 1.33, 95 per cent c.i. 0.80 to 2.23) also failed to be a prognostic factor for long-term survival when excluding those not surviving the first 90 days after hepatectomy. Increasing age, gallbladder cancer, and multivisceral resection, on the other hand, were all significantly associated with worse OS beyond 90 days. Survival according to PHLF of the 761 patients that lived beyond 90 days after hepatectomy is illustrated in *Fig. 2a,b*.

## Discussion

In this bi-institutional cohort of major hepatectomies, clinically relevant PHLF (grade B and C) was found to increase the risk of 90-day mortality and demonstrated a negative impact on long-term survival, while ISGLS grade A PHLF did not. However, the negative impact of PHLF grade B/C on long-term survival disappeared in patients surviving the first 90 days postoperatively.

The introduction of the ISGLS definition improved both the clinical and academic assessment of PHLF; however, divergence exists in reporting PHLF where some studies report PHLF as grade A–C^8,20^, whereas several other studies utilized the term ‘clinically relevant PHLF’ for ISGLS grade B/C^21–25^. The latter approach is clinically meaningful, as PHLF grade B and C both require treatment changes, whereas grade A is merely marked by deviating laboratory values. The results of the present study support a differentiation between grade A and grade B/C in terms of both short- and long-term survival.

PHLF is a feared complication in the early postoperative interval, particularly as causal treatment is not available. Liver failure arises when residual hepatocyte function is insufficient, mostly due to a small FLR, and when liver regeneration cannot compensate the loss of liver tissue ^26^. A recent review assumes an overall incidence of PHLF of around 8–12 per cent and highlights the extent of liver resection as a known risk factor^27^. In this study, 27 per cent of all patients were diagnosed with any ISGLS grade of PHLF and 13 per cent with clinically relevant PHLF, which is in line with earlier reports on major hepatectomies^6,7,28,29^. Consistent with previous studies, the presence of cirrhosis, type of primary liver malignancy, and the extent of liver resection were found to be associated with the risk of clinically relevant PHLF^30^.

In recent publications, postoperative 90-day mortality after major hepatectomy ranges from 2.3 to 9 per cent ^4,29,31–33^. In a Swedish nationwide analysis, 41 per cent of 90-day mortality after major and minor liver resection was related to PHLF^1^. In the present study, clinically relevant PHLF yielded a 90-day mortality rate of 21 per cent, which was considerably more than the 4 per cent in PHLF grade A. In the study by Reissfelder *et al*., which included both minor and major hepatectomies, a discrepancy in survival between grade A PHLF and grade B and C became obvious^6^. In that study, there were no postoperative deaths in grade A, whereas grade B and C liver failure were associated with a mortality rate of 13 per cent and 54 per cent respectively^6^. Sultana *et al*. similarly reported an increase in 90-day mortality from 0 per cent to 11 per cent and 89 per cent in grade A, B, and C PHLF^34^. Shehta *et al*. found a significant difference in OS between the ISGLS grades with worse survival in grade B/C^35^. In this study, both clinically relevant PHLF and PHLF defined by the ‘50–50 criteria’ proved to be feasible predictors of an increased risk of 90-day mortality, whereas ISGLS grade A liver failure was not associated with increased risk of postoperative death in the multivariable logistic model. In contrast, Birgin *et al*. reported an association of grade A PHLF with increased mortality^36^.

Although this study focused primarily on the different grades of PHLF and their impact on short- and long-term survival, some interesting results emerged from the sub-analysis on PHLF and postoperative morbidity. Major morbidity, defined as the highest Clavien–Dindo complication grade, was significantly more common in patients with PHLF grade A compared with those not developing PHLF, but that did not translate into a significant impact on mortality. The comprehensive complication index (CCI) has been shown to provide a more accurate assessment of patient morbidity compared with Clavien–Dindo score, which only considers the single most severe complication experienced^7,37^, but the former was unfortunately not available for the present study. Calthorpe *et al*. on the other hand, used CCI and observed that median CCI increased with increasing ISGLS grade but only grades B and C were significantly associated with increased odds of a high index. Still, they could not find an association between ISGLS grade A and high CCI or 90-day mortality. In the study of Calthorpe, the median CCIs observed in both grades B and C PHLF were substantially higher, suggesting that ISGLS grades B and C accumulate several major complications that together result in high CCI, whereas the highest Clavien–Dindo score may not differ significantly from ISGLS grade A, and that the quantity makes the difference^7^. This could possibly explain why although patients with ISGLS grade A have more major complication than those not suffering from PHLF, it does not impact mortality. There is, nevertheless, increasing evidence to differentiate between grade A and B/C liver failure regarding both morbidity and mortality, not least supported by the above-mentioned multicentric study focusing on major hepatectomies^7^.

Interestingly, in the present study, patients with PHLF according to the ISGLS definition (grades A–C) had a 90-day mortality of only 12.4 per cent (27 of 218 patients) compared with 20 per cent in the study by Reissfelder *et al*.^6^. Similarly, patients fulfilling the ‘50–50 criteria’ for PHLF had a 90-day mortality rate of only 31 per cent, despite that the definition is based on the prediction of a higher than 50 per cent risk of postoperative 60-day mortality^3^. The ‘50–50 criteria’ were published in 2005 and the improved postoperative outcome in the present study may be a result of a general better patient selection, improved operative techniques, and postoperative care.

While the early postoperative risk for mortality in patients with PHLF is well established, long-term results on survival are scarce and controversial. Some have found ISGLS PHLF to be associated with shorter OS but without analysing clinically relevant PHLF separately or analysing PHLF in univariable models only^8,28,31,35^. Contradictory, others did not find a significant impact of PHLF on long-term survival^9^. As PHLF definitions aim to identify patients at risk for early postoperative mortality the association with long-term survival remains unclear. Due to a high rate of postoperative mortality after PHLF, survival beyond the 90-day interval must be evaluated separately to identify possible long-term effects of PHLF, as previous studies have shown^9,28,35^. Consequently, when excluding the patients that died within 90 days after major hepatectomy in the present study, neither ISGLS grade A nor clinically relevant PHLF were associated with impaired long-term survival. Additionally, PHLF according to the ‘50–50 criteria’ also failed to be prognostic for long-term survival among those surviving 90 days after hepatectomy. Whether this is a true finding or a result of small sample size, cannot be elucidated from this study. A possible explanation might be that these criteria are feasible to identify short-term risks but survivors beyond 90 days after surgery do recover and have an equal survival as patients without PHLF.

Limitations of the present study are mainly related to its retrospective study design and the selection bias introduced by focusing only on major hepatectomies. Both primary and secondary liver malignancies and benign conditions were included and each of these diagnoses are associated with quite different postoperative survival rates that could influence the results. It cannot be excluded that the underlying malignant diagnoses are the determinates of the results rather than PHLF itself, although PHLF grade B/C was found to be an independent predictor of OS when adjusting for multiple factors, including primary tumour origin. Furthermore, some patient characteristics are lacking, for example data on acute kidney injury and liver function in the longer run, which could affect postoperative treatment and survival^38^. As PHLF is a leading cause of life-threatening complications after major hepatectomy, various methods have been proposed to determine the risk of PHLF before surgery^39^. The predictive potential of combined aspartate aminotransferase/platelet ratio index and albumin–bilirubin grade for grade C PHLF was recently shown^40^, but data to validate the combined score were not available in the present study. Futhermore, sufficient data on the FLR size were missing and not possible to include in statistical analyses. The results of this study are not intended to change the clinical management of patients with deviating liver function parameters directly, as the definition of PHLF according to the ISGLS can only be applied retrospectively. Documenting the occurrence of grade A PHLF will still be relevant, as it, according to the present study, affects major co-morbidity and might have other clinical implications, such as an effect on duration of hospital stay as previously shown by Birgin *et al*.^36^, which needs further evaluation in future studies. Given the low incidence of PHLF, future collaboration with multiple institutions is strongly encouraged.

The results of this study support a differentiation between different grades of PHLF according to the ISGLS criteria in future studies on mortality, not only in view of postoperative clinical management, but also in terms of long-term survival. ISGLS grade A PHLF was neither associated with increased risk of 90-day mortality, nor long-term mortality and should therefore not be grouped together with grades B and C when studying mortality. Although ISGLS criteria were created to describe mortality in relation to PHLF, this study showed that when analysing different ISGLS grades in relation to postoperative morbidity, even grade A has clinical implications.

## Supplementary Material

zrac097_Supplementary_Data

## Data Availability

Raw data were generated at Karolinska University Hospital and Linköping University Hospital. Derived data supporting the findings of this study are available from the corresponding author J.E. on request after appropriate ethical approval.
