# Supplementary material for: Impact of post-hepatectomy liver failure on morbidity and short- and long-term survival after major hepatectomy
Source: BJS Open. 2022 Jul 16;6(4):zrac097. doi: 10.1093/bjsopen/zrac097 (PMC9291378; doi:10.1093/bjsopen/zrac097)
Supplement: zrac097_Supplementary_Data [file zrac097_supplementary_data.docx]

Table S1. The association between pre- and intraoperative clinical and tumour characteristics and post-hepatectomy liver failure defined as International Study Group of Liver Surgery grade B or C.

|  | Univariable analysis | | Multivariable analysis | |
| --- | --- | --- | --- | --- |
|  | OR (95% CI) | *p* | OR (95% CI) | *p* |
| Age ≥65 | 0.94 (0.62-1.43) | 0.783 |  |  |
| Male sex | 1.35 (0.88-2.06) | 0.167 |  |  |
| Diabetes | 1.97 (1.17-3.33) | 0.011 | 1.76 (0.75-4.12) | 0.195 |
| Pulmonary disease | 0.43 (0.17-1.10) | 0.079 | 0.17 (0.03-0.83) | **0.028** |
| Cardiovascular disease | 1.28 (0.84-1.93) | 0.252 |  |  |
| Cirrhosis | 7.04 (3.21-15.45) | <0.001 | 45.31 (6.56-313.1) | **<0.001** |
| Portal vein occlusion | 2.72 (1.64-4.51) | <0.001 | 0.60 (0.25-1.43) | 0.247 |
| Tumour type |  |  |  |  |
| CRLM | Ref |  | Ref |  |
| HCC | 0.79 (0.30-2.07) | 0.625 | 0.16 (0.20-1.21) | 0.076 |
| pCCC | 3.42 (1.97-5.95) | <0.001 | 3.73 (1.37-10.18) | **0.010** |
| iCCC | 0.56 (0.19-1.61) | 0.283 | 1.24 (0.29-5.22) | 0.769 |
| Gallbladder cancer | 10.94 (4.29-27.92) | <0.001 | 6.39 (1.11-36.77) | **0.038** |
| Other malignant tumours | 0.64 (0.22-1.85) | 0.410 | 0.50 (0.10-2.48) | 0.392 |
| Benign tumours | 1.49 (0.73-3.05) | 0.272 | 1.15 (0.36-3.75) | 0.812 |
| Preoperative bilirubin ≥35μmol/L | 2.5 (0.86-7.26) | 0.092 | 0.60 (0.08-4.33) | 0.609 |
| Preoperative albumin ≥30 g/L | 0.48 (0.22-1.03) | 0.061 | 0.69 (0.25-1.92) | 0.479 |
| Preoperative INR ≥1.3 | 1.30 (0.43-3.90) | 0.643 |  |  |
| Extent of liver resection |  |  |  |  |
| Left hemihepatectomy | Ref |  | Ref |  |
| Left trisectionectomy | 1.61 (0.35-7.42) | 0.541 | 0.42 (0.06-3.01) | 0.389 |
| Right hemihepatectomy | 4.53 (1.61-12.77) | 0.004 | 5.35 (1.37-20.84) | **0.016** |
| Right trisectionectomy | 11.26 (3.93-32.24) | <0.001 | 6.99 (1.74-28.06) | **0.006** |
| Pringle manoeuvre | 2.23 (1.22-4.07) | 0.009 | 1.53 (0.60-3.91) | 0.370 |
| Prolonged operation time ≥240 min | 4.57 (2.50-8.36) | <0.001 | 3.72 (1.59-8.69) | **0.002** |
| Multivisceral resection | 1.76 (0.85-3.65) | 0.126 |  |  |
| Blood transfusion | 3.19 (2.09-4.87) | <0.001 | 1.77 (0.92-3.42) | 0.089 |

OR, odds ratio; CRLM, colorectal cancer liver metastases; HCC, hepatocellular carcinoma; pCCC, perihilar cholangiocarcinoma; iCCC, intrahepatic cholangiocarcinoma; INR, International Normalized Ratio.

Table S2. The association between pre- and perioperative clinical factors and 90-day mortality.

|  | Univariable analysis | | Multivariable analysis | |
| --- | --- | --- | --- | --- |
|  | OR (95% CI) | *p* | OR (95% CI) | *p* |
| Age ≥65 | 2.19 (1.07-4.48) | 0.032 | 2.58 (1.08-6.14) | **0.032** |
| Male sex | 1.14 (0.59-2.21) | 0.696 |  |  |
| Diabetes | 3.72 (1.84-7.53) | <0.001 | 2.24 (0.89-5.68) | 0.088 |
| Pulmonary disease | 1.09 (0.38-3.15) | 0.875 |  |  |
| Cardiovascular disease | 2.01 (1.04-3.90) | 0.038 | 1.04 (0.44-2.47) | 0.925 |
| Cirrhosis | 5.09 (1.81-14.28) | 0.002 | 2.97 (0.66-13.37) | 0.157 |
| Portal vein occlusion | 1.87 (0.83-4.21) | 0.129 |  |  |
| Tumour type |  |  |  |  |
| CRLM | Ref |  | Ref |  |
| HCC | 1.58 (0.44-5.72) | 0.483 | 0.95 (0.17-5.14) | 0.950 |
| pCCC | 3.71 (1.57-8.74) | 0.003 | 3.71 (1.04-13.26) | **0.043** |
| iCCC | 0.94 (0.21-4.28) | 0.940 | 1.50 (0.29-7.68) | 0.629 |
| Gallbladder cancer | 16.78 (5.74-49.01) | <0.001 | 8.82 (1.77-43.97) | **0.008** |
| Other malignant tumours | 1.07 (0.24-4.88) | 0.926 | 1.68 (0.33-8.62) | 0.536 |
| Benign tumours | 0.41 (0.05-3.18) | 0.394 | 0.28 (0.03-2.69) | 0.273 |
| Preoperative bilirubin ≥35 μmol/L | 2.37 (0.52-10.79) | 0.263 |  |  |
| Preoperative albumin ≥30 g/L | 0.49 (0.16-1.49) | 0.206 |  |  |
| Preoperative INR ≥1.3 | 1.70 (0.38-7.53) | 0.486 |  |  |
| Extent of liver resection |  |  |  |  |
| Left hemihepatectomy | Ref |  | Ref |  |
| Left trisectionectomy | 6.59 (0.67 (64.64) | 0.106 | 3.75 (0.35-40.59) | 0.277 |
| Right hemihepatectomy | 6.57 (0.87-49.41) | 0.067 | 4.38 (0.52-36.67) | 0.173 |
| Right trisectionectomy | 11.87 (1.54-91.48) | 0.018 | 2.87 (0.32-25.41) | 0.343 |
| Pringle manoeuvre | 1.26 (0.43-3.66) | 0.671 |  |  |
| Prolonged operation time ≥240 min | 3.33 (1.38-8.07) | 0.008 | 1.36 (0.48-3.84) | 0.556 |
| Multivisceral resection | 2.41 (0.90-6.47) | 0.081 | 1.87 (0.51-6.82) | 0.345 |
| Blood transfusion | 2.84 (1.47-5.49) | 0.002 | 1.57 (0.71-3.46) | 0.262 |
| PHLF ISGLS |  |  |  |  |
| No PHLF | Ref |  | Ref |  |
| A | 2.36 (0.80-6.91) | 0.119 | 2.43 (0.77-7.65) | 0.128 |
| B and C | 14.07 (6.58-30.10) | <0.001 | 7.26 (2.90-18.17) | **<0.001** |

OR, odds ratio; CRLM, colorectal cancer liver metastases; HCC, hepatocellular carcinoma; pCCC, perihilar cholangiocarcinoma; iCCC, intrahepatic cholangiocharcinoma; INR, International Normalized Ratio; PHLF, post-hepatectomy liver failure; ISGLS, International Study Group of Liver Surgery. Univariable analysis of PHLF according to Balzan et al. yielded an OR of 11.99 (95% CI 5.35-26.87), p<0.001. When replacing PHLF according to ISGLS to that of Balzan et al. in the multivariable model, the OR was 6.73 (95% CI 2.41-18.79), p<0.001.

Table S3. Univariable and multivariable analysis for overall survival in patients undergoing major hepatectomy

|  | Univariable | | Multivariable | |
| --- | --- | --- | --- | --- |
|  | HR (95% CI) | *p* | HR (95% CI) | *p* |
| **Patient characteristics** |  |  |  |  |
| Age, years | 1.02 (1.01-1.03) | <0.001 | 1.01 (0.99-1.02) | 0.119 |
| Male sex | 1.16 (0.96-1.39) | 0.123 |  |  |
| Diabetes | 1.16 (0.90-1.50) | 0.262 |  |  |
| Pulmonary disease | 1.18 (0.88-1.58) | 0.268 |  |  |
| Cardiovascular disease | 1.28 (1.06-1.53) | 0.009 | 1.21 (0.90-1.63) | 0.203 |
| **Tumour/liver characteristics** |  |  |  |  |
| Cirrhosis | 1.16 (0.71-1.92) | 0.554 |  |  |
| Portal vein occlusion | 1.66 (1.30-2.13) | <0.001 | 1.63 (1.14-2.33) | **0.007** |
| Tumour type |  |  |  |  |
| CRLM | Ref |  | Ref |  |
| HCC | 0.75 (0.51-1.10) | 0.137 | 0.68 (0.42-1.10) | 0.118 |
| pCCC | 1.57 (1.21-2.05) | 0.001 | 1.22 (0.80-1.86) | 0.352 |
| iCCC | 1.48 (1.10-2.00) | 0.010 | 1.04 (0.66-1.65) | 0.860 |
| Gallbladder cancer | 5.60 (3.48-9.01) | <0.001 | 7.28 (3.26-16.28) | **<0.001** |
| Other malignant tumours | 0.51 (0.34-0.79) | 0.002 | 0.34 (0.16-0.73) | **0.005** |
| Benign tumours | 0.14 (0.07-0.27) | <0.001 | 0.13 (0.05-0.35) | **<0.001** |
| **Preoperative tests** |  |  |  |  |
| Preoperative bilirubin ≥35μmol/L | 1.20 (0.66-2.18) | 0.556 |  |  |
| Preoperative albumin ≥30 g/L | 0.66 (0.44-1.01) | 0.056 | 0.83 (0.52-1.30) | 0.400 |
| Preoperative INR ≥1.3 | 1.14 (0.67-1.94) | 0.631 |  |  |
| **Intraoperative factors** |  |  |  |  |
| Extent of liver resection |  |  |  |  |
| Left hemihepatectomy | Ref |  | Ref |  |
| Left trisectionectomy | 1.66 (1.14-2.42) | 0.008 | 1.21 (0.75-1.95) | 0.430 |
| Right hemihepatectomy | 1.07 (0.82-1.40) | 0.627 | 0.80 (0.55-1.16) | 0.237 |
| Right trisectionectomy | 1.71 (1.27-2.31) | <0.001 | 1.07 (0.68-1.69) | 0.776 |
| Pringle manoeuvre | 1.10 (0.79-1.51) | 0.586 |  |  |
| Prolonged operation time ≥240 min | 1.34 (1.11-1.63) | 0.003 | 0.90 (0.67-1.21) | 0.491 |
| Multivisceral resection | 1.49 (1.05-2.13) | 0.027 | 1.77 (1.01-3.09) | **0.046** |
| Blood transfusion | 1.29 (1.06-1.56) | 0.010 | 1.26 (0.95-1.66) | 0.105 |
| **Postoperative factors** |  |  |  |  |
| Biliary leakage^a^ (0 vs A-C) | 1.29 (1.04-1.59) | 0.019 | 1.11 (0.80-1.54) | 0.534 |
| PHLF ISGLS |  |  |  |  |
| No PHLF | Ref |  | Ref |  |
| A | 1.22 (0.94-1.58) | 0.124 | 1.25 (0.87-1.81) | 0.229 |
| B and C | 1.93 (1.50-2.47) | <0.001 | 1.90 (1.32-2.71) | **<0.001** |

HR, hazard ratio; CRLM, colorectal cancer liver metastases; HCC, hepatocellular carcinoma; pCCC, perihilar cholangiocarcinoma; iCCC; intrahepatic cholangiocarcinoma; INR, International Normalized Ratio ; PHLF, post-hepatectomy liver failure; ISGLS, International Study Group of Liver Surgery.

^a^ Definition and grading by the ISGLS.

Univariable analysis of PHLF according to Balzan et al. yielded an HR of 2.09 (95% CI 1.41-3.08), p<0.001. When replacing PHLF according to ISGLS to that of Balzan et al. in the multivariable model, the HR was 1.72 (95% CI 1.09-2.72), p=0.020

Table S4. Univariable and multivariable analysis for overall survival in patients undergoing major hepatectomy surviving 90 days postoperatively (n=761).

|  | Univariable | | Multivariabe | |
| --- | --- | --- | --- | --- |
|  | HR (95% CI) | *p* | HR (95% CI) | *p* |
| **Patient characteristics** |  |  |  |  |
| Age, years | 1.02 (1.01-1.03) | <0.001 | 1.02 (1.01-1.03) | 0.002 |
| Male sex | 1.15 (0.95-1.39) | 0.152 |  |  |
| Diabetes | 1.02 (0.77-1.35) | 0.899 |  |  |
| Kidney failure | 1.37 (0.80-2.33) | 0.248 |  |  |
| Pulmonary disease | 1.22 (0.90-1.65) | 0.207 |  |  |
| Cardiovascular disease | 1.23 (1.02-1.49) | 0.033 | 1.01 (0.81-1.25) | 0.934 |
| **Tumour/liver characteristics** |  |  |  |  |
| Cirrhosis | 0.95 (0.53-1.68) | 0.854 |  |  |
| Portal vein occlusion | 1.64 (1.26-2.13) | <0.001 | 1.41 (1.05-1.90) | 0.024 |
| Tumour type |  |  |  |  |
| CRLM | Ref |  | Ref |  |
| HCC | 0.73 (0.50-1.09) | 0.123 | 0.71 (0.47-1.06) | 0.094 |
| pCCC | 1.44 (1.09-1.91) | 0.011 | 1.07 (0.75-1.51) | 0.720 |
| iCCC | 1.52 (1.13-2.07) | 0.006 | 1.31 (0.94-1.82) | 0.114 |
| Gallbladder cancer | 4.44 (2.47-7.99) | <0.001 | 3.26 (1.69-6.28) | <0.001 |
| Other malignant tumours | 0.49 (0.31-0.76) | 0.002 | 0.50 (0.32-0.79) | 0.003 |
| Benign tumours | 0.13 (0.06-0.26) | <0.001 | 0.15 (0.07-0.30) | <0.001 |
| **Preoperative tests** |  |  |  |  |
| Preoperative bilirubin ≥20 | 1.09 (0.56-2.11) | 0.807 |  |  |
| Preoperative albumin ≤30 | 0.70 (0.44-1.10) | 0.122 |  |  |
| Preoperative INR >1.3 | 1.09 (0.61-1.93) | 0.780 |  |  |
| **Intraoperative factors** |  |  |  |  |
| Extent of liver resection |  |  |  |  |
| Left hemihepatectomy | Ref |  | Ref |  |
| Left trisectionectomy | 1.60 (1.09-2.35) | 0.016 | 1.12 (0.74-1.69) | 0.581 |
| Right hemihepatectomy | 0.99 (0.75-1.30) | 0.946 | 0.79 (0.59-1.07) | 0.130 |
| Right trisectionectomy | 1.58 (1.16-2.15) | 0.004 | 1.09 (0.76-1.56) | 0.652 |
| Pringle manoeuvre | 1.08 (0.77-1.51) | 0.666 |  |  |
| Prolonged operation time ≥240 min | 1.26 (1.03-1.53) | 0.026 | 1.09 (0.87-1.36) | 0.470 |
| Multivisceral resection | 1.39 (0.95-2.04) | 0.091 | 1.52 (1.01-2.30) | 0.047 |
| Blood transfusion | 1.19 (0.98-1.46) | 0.086 | 1.19 (0.96-1.47) | 0.115 |
| **Postoperative factors** |  |  |  |  |
| Biliary leakage^a^ (0 vs A-C) | 1.30 (1.04-1.62) | 0.020 | 1.31 (1.03-1.65) | 0.027 |
| PHLF according to ISGLS |  |  |  |  |
| No PHLF | Ref |  | Ref |  |
| A | 1.18 (0.91-1.54) | 0.216 | 1.28 (0.97-1.70) | 0.081 |
| B and C | 1.46 (1.10-1.95) | 0.009 | 1.30 (0.95-1.77) | 0.104 |

HR, hazard ratio; CRLM, colorectal cancer liver metastases; HCC, hepatocellular carcinoma; pCCC, perihilar cholangiocarcinoma; iCCC; intrahepatic cholangiocarcinoma; INR, International Normalized Ratio ; PHLF, post-hepatectomy liver failure; ISGLS, International Study Group of Liver Surgery.

^a^ Definition and grading by the ISGLS.

Univariable analysis of PHLF according to Balzan et al. yielded an HR of 1.35 (95% CI 0.82-2.22), p=0.174. When replacing PHLF according to ISGLS to that of Balzan et al. in the multivariable model, the HR was 1.33 (95% CI 0.80-2.23), p=0.271.
